# Economic hardship associated with managing chronic illness: a qualitative inquiry

**DOI:** 10.1186/1472-6963-9-182

**Published:** 2009-10-09

**Authors:** Yun-Hee Jeon, Beverley Essue, Stephen Jan, Robert Wells, Judith A Whitworth

**Affiliations:** 1 The Australian Primary Health Care Research Institute, The College of Medicine, Biology and Environment, The Australian National University, Building 62, Mills Rd, Canberra, ACT 0200, Australia; 2 The Faculty of Nursing and Midwifery, The University of Sydney, 88 Mallett Street, Camperdown, NSW 2050, Australia; 3 Menzies Centre for Health Policy, The University of Sydney, Victor Coppleson Bldg (D02), Camperdown, NSW 2006, Australia; 4 The George Institute for International Health, Level 10 King George V Building, Royal Prince Alfred Hospital, Missenden Road, Camperdown, NSW 2050, Australia; 5 Menzies Centre for Health Policy, The College of Medicine, Biology and Environment, The Australian National University, Building 131, Garran Road, Canberra, ACT 0200, Australia; 6 The John Curtin School of Medical Research, The College of Medicine, Biology and Environment, The Australian National University, Building 131, Garran Road, Canberra, ACT 0200, Australia

## Abstract

**Background:**

Chronic illness and disability can have damaging, even catastrophic, socioeconomic effects on individuals and their households. We examined the experiences of people affected by chronic heart failure, complicated diabetes and chronic obstructive pulmonary disease to inform patient centred policy development. This paper provides a first level, qualitative understanding of the economic impact of chronic illness.

**Methods:**

Interviews were conducted with patients aged between 45 and 85 years who had one or more of the index conditions and family carers from the Australian Capital Territory and Western Sydney, Australia (n = 66). Content analysis guided the interpretation of data.

**Results:**

The affordability of medical treatments and care required to manage illness were identified as the key aspects of economic hardship, which compromised patients' capacity to proactively engage in self-management and risk reduction behaviours. Factors exacerbating hardship included ineligibility for government support, co-morbidity, health service flexibility, and health literacy. Participants who were on multiple medications, from culturally and linguistically diverse or Indigenous backgrounds, and/or not in paid employment, experienced economic hardship more harshly and their management of chronic illness was jeopardised as a consequence. Economic hardship was felt among not only those ineligible for government financial supports but also those receiving subsidies that were insufficient to meet the costs of managing long-term illness over and above necessary daily living expenses.

**Conclusion:**

This research provides insights into the economic stressors associated with managing chronic illness, demonstrating that economic hardship requires households to make difficult decisions between care and basic living expenses. These decisions may cause less than optimal health outcomes and increased costs to the health system. The findings support the necessity of a critical analysis of health, social and welfare policies to identify cross-sectoral strategies to alleviate such hardship and improve the affordability of managing chronic conditions. In a climate of global economic instability, research into the economic impact of chronic illness on individuals' health and well-being and their disease management capacity, such as this study, provides timely evidence to inform policy development.

## Background

The prevention, management and treatment of chronic, non-communicable illness are major issues facing governments in the 21^st ^Century [1,2]. In 2002 approximately 59 per cent of global death was attributable to chronic, non-communicable diseases and the toll is projected to increase to 66 per cent by 2030 [3]. The estimates are markedly higher for developed countries, where almost all deaths (86%) are attributable to this disease category [4]--in Australia around 80% of the total burden of illness and injury is accounted for by chronic, non-communicable diseases [5]. In 2004, 77% of the Australian population reported at least one long-term condition and more than 80% of those in the older age groups had three or more long-term conditions [5]. In a similar period, over 1.4 million older people in Australia (56% of the older population) had at least one form of disability, possibly associated with chronic health conditions that restricted their everyday activities [6].

### Economic consequences of chronic illness

Chronic illness and disability can have damaging, even catastrophic, socioeconomic effects on individuals and their households. In Australia in 2003-4, 2.1 million people received government disability and sickness related payments, including over 400,000 recipients of carer allowances, carer payments and spouse pensions paid to those caring for a spouse with illness [7]. Furthermore, the number of people receiving these payments has increased steadily in recent years--a pattern observed across all Organisation for Economic Co-operation and Development (OECD) countries, with disability and sickness benefit payments now accounting for a significant proportion of national income [7].

Internationally, there is a well established correlation between low socioeconomic circumstances, poor health and well-being [8] and an increased risk of mortality among those with serious health conditions [9]. Palmer et al. report that 3 in 10 disabled people live below the poverty line in OECD countries [10]. However, in high income countries, such as Australia, cushioned with publicly funded health care and social security arrangements, the economic effects of long-term illness are not as immediately obvious.

Terminology describing and measuring economic hardship, or economic consequences, of chronic illness varies. One approach is to measure at the macroeconomic level, direct costs (i.e., costs to the health sector), indirect costs (i.e., lost production due to disability/illness and premature death), and intangible costs (i.e., psychological consequences of illness) [11,12]. Using this macroeconomic analysis, the costs of chronic illness and their risk factors range globally from 0.02 per cent to 6.77 per cent of Gross Domestic Product (GDP) [12].

The alternative approach focuses on the impact of illness at a microeconomic level (i.e., individuals and households). This characterises much of the research conducted in Australia, particularly in the social welfare literature, which shows a significant correlation between financial stress, disability and poor physical and mental health [13,14]; and between poverty rates (defined in narrow income terms) and the disability (handicap, a long-term condition or chronic illness) [15]. In his recent study on the costs of disability, derived from the analysis of the 1998-99 Household Expenditure Survey (HES) data, Saunders [15] estimates the costs of disability to be approximately 29 per cent of household income, which increases up to 49 per cent for those with a severe restriction; and suggests the poverty rate among those with a disability can be six times higher than those without a disability. Saunders argues that existing income support arrangements for people with a disability is far from sufficient to maintain a minimum standard of living.

During a preliminary literature review we found no Australian qualitative research that examined the economic impact of chronic illness. The most relevant investigation of this topic is the Senate Community Affairs Committee's recent report on cost of living pressures for older Australians [16]. The report provides timely insights into the types of economic hardship that older people and their households face in everyday life as a result of illness.

### Snapshot of the Australian health and social welfare system

Australia has a national system of health insurance, known as Medicare, which guarantees free public hospital treatment based on clinical need for all Australians. Medicare is operated by the Australian (national) Government. Public hospitals are operated by state/territory governments and the Australian Government meets a share of the public hospital costs in return for the Medicare guarantee. In addition Medicare rebates some of the costs of medical and some nursing and allied health services provided by practitioners in private settings on a fee-for-service basis. This arrangement extends to in-hospital services provided on a private basis. Primary care services provided by general practitioners are funded through this latter arrangement. Providers may set their own fees, with rebates attached to a government determined schedule. Patients may then be required to meet the difference between the provider's fee and the rebate, however many services are provided at no charge to the patient. The cost of certain prescribed medications is also subsidised under Medicare [17,18].

The social welfare system is administered separately from Medicare. Persons in receipt of certain welfare payments (e.g., age or disability pensions) receive higher rebates for medical services and pay less for prescribed medications. Medicare covers the costs of professional services only and does not include treatment supports (e.g., home oxygen, aids and appliances); state governments provide some or all of these services, usually with a means test [19].

As a result of these complex arrangements, patients often find themselves faced with out-of-pocket expenses, receive different community supports depending on where they live and often find that important medical support for their treatment is not available.

### The Serious and Continuing Illness Policy and Practice Study (SCIPPS)

SCIPPS was designed to develop policy and health system interventions to support the provision of optimal care for patients with chronic illness and their carers [20]. SCIPPS focuses on complicated diabetes, chronic heart failure (CHF) and chronic obstructive pulmonary disease (COPD) as the three most common, costly and consistent conditions that have discrete intervention points along the continuum of care and strong evidence of successful intervention.

One of the key objectives of SCIPPS is to engage health care providers, users, communities, funders and policy makers in developing best practice health care arrangements for chronic disease care through policy and system interventions. Initially, a qualitative study was conducted to develop a comprehensive understanding of the experiences of patients and carers affected by the index conditions to inform patient-centred policy development. The analysis indicated that people affected by chronic illnesses act purposefully to balance their quality of life with the impact of their diseases, which we called "balancing life and illness" (BLI) [21]. While experiencing a state of tension between the desire to lead a normal life and the constraints imposed by their illnesses, patients and carers had to weigh competing priorities and make decisions about courses of action to strike a balance between the management of their chronic condition and activities that are important to them in getting on with their lives.

The purpose of this paper is to report the findings of the secondary analysis of the qualitative data focusing on the economic hardship--recognised as one of the key challenges that interfered with the process of BLI, its meaning as well as the key factors that contribute to and exacerbate economic hardship, based on the experiences of those who are affected by chronic illness. Economic hardship in this paper is defined as perceived economic difficulties that arise as a result of chronic illness and influence the way in which people affected by illness live and manage their conditions.

## Methods

Data collection occurred between March 2007 and January 2008 in the Australian Capital Territory (ACT) and Western suburbs of Sydney. Semi-structured, in-depth interviews were conducted with patients and with carers; each interview running between 45 and 90 minutes. Patients and carers then completed a 10 minute demographic survey, which contained information about the patient's health conditions and health care encounters. During the survey, participants were also asked about financial issues including: their current work status, whether or not they are experiencing financial difficulties or under financial pressure, and whether they receive any government financial benefits.

Patients were recruited through referrals from general practices, local hospitals, community health services, specialist clinics, health care consumer organisations, and Aboriginal health services located in the two regions. Patients were included in the study if they were aged between 45 and 85 years, had one or more of the three index conditions that required long term medical interventions and were deemed to be seriously ill according to their referring clinician. Purposive sampling was used to achieve variation in patient characteristics including age, diagnosis, geographical location, Indigenous status and cultural and linguistic background. Carers were defined as the most centrally involved family member or close friend providing practical and emotional assistance to the patient. Carers were recruited using convenience sampling, primarily through their care recipient (9 of 14 carers) and through the health professionals at the centres listed above. Sixty-six of 80 patients and carers approached agreed to participate in the study (82.5% response rate).

Ethics approval was granted by the Australian National University Human Research Ethic Committee, the ACT Health Human Research Ethics Committee, the University of Sydney Human Research Ethics Committee and the Sydney West Area Health Service Human Research Ethics Committee. All individuals gave informed consent prior to participating.

The patient and carer interviews began with a question asking the participant: 'what it is like to live (or care for someone) with chronic illness', followed by questions about the challenging and helpful aspects of their experience. The research team judged sufficient data had been gathered when interviews no longer provided new insights or ideas central to the patients' and carers' experience, indicating data saturation had occurred [22].

All interviews were electronically recorded and transcribed verbatim. The data were analysed using qualitative content analysis [23], assisted by a computerised qualitative data analysis program, QSR NVivo7 [24]. Using the NVivo 7 matrix function, the relationships between the key issues of economic hardship and demographic and clinical characteristics were examined. Descriptive analysis of the survey data was undertaken using SPSS version 15 [25].

Lincoln and Guba's [26] criteria were used to maximise the reliability and validity (i.e., the credibility, transferability, dependability, and confirmability) of the data and the following analysis including: extensive researcher training and practice in interview skills and data analysis, data management using NVivo7 [24], use of a pilot to assure adequacy of the data collection tool and recruitment strategies, development of protocols and a coding scheme, establishment of inter-coder reliability, and examination of qualitative data against relevant survey data.

## Results

### Characteristics of participants (survey data)

Fifty-two patients and fourteen carers participated in the study. Most of them were older than 65 years (n = 42)--on average 69 years old for patients and 63 years old for carers; were born in Australia, New Zealand or UK (n = 50); had more than a decade-long history of chronic illness (mean = 16.5 years); and had monthly or more frequent contact with general practitioners (GP). Fifty-nine participants (89.4%) were not in paid employment (i.e., completely retired/on pension, or unemployed). A similar number of participants (n = 58) were receiving some form of government financial benefits at the time of the interview. Detailed characteristics of the participants are summarised in Tables 1 and 2.

**Table 1:** Characteristics of study participants

**Categories**	**Sub-categories**	**Patient (n = 52)**	^**§**^**Carer (n = 14)**
**Residence**	Australian Capital Territory	26 (50%)	6 (43%)
	Sydney West	26 (50%)	8 (57%)

**Gender**	Male	28 (54%)	1 (7%)
	Female	24 (46%)	13 (93%)

**Age**	Up to 64 years	17 (33%)	7 (50%)
	65 years and over	35 (67%)	7 (50%)

**Special groups**	^α^Culturally & linguistically diverse background	11 (21%)	5 (36%)
	Indigenous Australians	7 (14%)	0

**Marital Status**	Married/de facto/living with a partner	29 (56%)	13 (93%)

^**β**^**Diagnosis**	Type 2 diabetes	27 (52%)	*7 (50%)
	Chronic heart failure	20 (38%)	*4 (29%)
	Chronic obstructive pulmonary disease	17 (33%)	*7 (50%)
	More than one index condition	11 (21%)	*3 (21%)
	Average length of illness (years)	16.5	*21.4
	Other co-morbid conditions	43 (87%)	*11 (86%)

**Visit to GP**	Monthly or more often	35 (67%)	*12 (86%)
	Bi-monthly or less frequently	17 (33%)	*2 (14%)

**Family Carer**	Have a family carer	^#^22 (42%)	Non applicable
	Average length of caring (years)	^#^14.7	12.5
	Carer receives financial assistance	^#^5 (23%)	5 (36%)
	Carer receives informal support	^#^14 (64%)	9 (64%)

^α ^Born in one of the following countries: Finland, France, Germany, Hungary, Turkey, Syria, South Africa, Philippines, Malaysia, Samoa, Hong Kong, China

^β ^The total number combines more than 52 because ten patients had two of the index conditions and one patient had all three conditions

^§ ^Nine carers were spouses or offspring of the patients interviewed

* Denotes the carer's account about the patient's condition and management

^# ^Denotes the patient's account about the carer

**Table 2:** Participant characteristics associated with economic status

**Characteristics**	**Patients (n = 52)**	**Carers (n = 14)**
**Work Status**		
Full-time or part-time employed	2 (3.8%)	2 (14.2%)
Self-employed	2 (3.8%)	0
Partially retired	1 (1.9%)	0
Completely retired pensioner	40 (76.9%)	7 (50%)
Disabled/sickness pension	6 (11.5%)	0
Looking after home/family	1 (1.9%)	5 (35.7%)

**Government Financial Benefits (multiple responses)**		
Disability Support Pension	10 (9.7%)	0
Mobility Allowance	1 (0.9%)	0
Health Care Card	12 (11.7%)	2 (7.4%)
Pensioner Concession Card	28 (27.2%)	6 (22.2%)
Unemployment Benefit	1 (0.9%)	0
Aged Pension	25 (24.3%)	6 (22.2%)
Sickness Allowance	1 (0.9%)	1 (3.7%)
Veteran's Affairs Disability Pension	3 (2.9%)	0
Commonwealth Seniors Card	18 (17.4%)	3 (11.1%)
None	4 (3.9%)	4 (14.8%)
Other (unable to describe)	0	5 (18.5%)

^**α**^**Experiencing financial difficulties (Yes)**	31 (60%)	11 (79%)

^α ^Whether or not participants perceived to have financial difficulties, or were under any financial pressure due to the costs associated with the chronic illness

Costs associated with the management of chronic illness was raised by eighty-two per cent of patients and carers (n = 54) as one of the key challenges they faced. Sixty-four per cent (n = 42) indicated that they experienced financial hardship, caused by the costs of illness management and caring responsibilities, which they believed negatively impacted on their quality of life.

### Economic consequences (matrix of the interview and survey data)

Content analysis of the interviews revealed that participants experienced the economic impact of chronic illness in terms of their ability to afford necessary medical care and treatments (*Affordability of treatment*) and recommended self-management activities and basic living costs (*Affordability of other things*). Table 3 illustrates the key characteristics and the frequency of participants who raised the affordability issues as negative factors during the interviews. When the two sites (Western Sydney and ACT) were compared, a considerably higher number of the ACT participants identified the affordability of treatment as a contributing factor in their economic hardship. More CHF and COPD patients reported economic hardship as a result of their illness than those with diabetes. Also, the majority of participants who were currently on medication, had co-morbidities and were not in paid employment reported that they had experienced economic hardship.

**Table 3:** Participant characteristics and affordability issues

**Characteristics** **(No of participants)**	^**α**^**Affordability of treatment** **(n = 28)**	^**β**^**Affordability of other things** **(n = 29)**	*Any-affordability **(n = 42)**	^**#**^**Both-affordability** **(n = 15)**
**ACT (32)**	17	13	22	8

**Sydney West (34)**	11	16	20	7

**Indigenous Australians (7)**	4	4	5	3

^**§**^**CALD (14)**	4	5	7	2

^**Δ**^**CHF (24)**	9	15	16	8

^**Δ**^**COPD (23)**	10	11	17	4

^**Δ**^**Diabetes (35)**	14	14	21	7

**Co-morbidity (49)**	16	22	28	10

**Female (37)**	16	15	23	8

**Male (29)**	12	14	19	7

^**Φ**^**On Medication (58)**	26	28	39	15

**Not in paid employment^ (59)**	25	26	38	13

^α ^Affordability of treatment included individuals' capacity to pay for medications, health check-ups, appointment with health professionals, other medical care expenses, etc.

^β ^Affordability of other things included individuals' capacity to pay for basic living expenses, healthy food, exercise and gym memberships, social activities and transport, etc.

* Participants who responded either in terms of affordability of 'treatment' or 'other things'

^# ^Participants who responded both in terms of affordability of 'treatment' and 'other things'

^§ ^Culturally & Linguistically Diverse background

^Δ ^Some participants have more than one index condition; the total is larger than the total sample size

^Φ ^Patients currently on medication for their chronic condition

^ Participants who were not in any form of paid employment at the time of the interview

Three key themes from the interviews are described in detail below.

### Affordability of treatment

Affordability of treatment referred to participant's ability to pay for any treatment, service and care required to manage their chronic conditions and its consequences. Patients and carers expressed concerns regarding ongoing financial pressures due to high costs involved in the treatment and management of chronic illness (i.e., out-of-pocket costs for medications, oxygen, regular check-ups, blood sugar level testing kits, specialist and other medical care), often accompanied by existing economic constraints and lack of support resources. For example,

*Our total income of meagre savings has been taken with payment for food, rent, and healthcare costs. These include a large bill each month for essential heart medicines, but also the costs of frequent consultations with GP and consultant cardiologists. These visits lead to the expense of oft-repeated laboratory tests, including vital INR readings, routine blood tests for digoxin levels, diabetes, ECGs, heart scans, a mechanical valve, and a few check-ups on the pacemaker*. (Man in his late sixties with CHF)

Financial pressures also arose from other treatment costs and the need to make home modifications or purchase necessary assistive equipment for disease management.

*You can't walk anywhere, it's taxis everywhere you want to go, with the oxygen cylinder, plus the cost of the oxygen and the oxygen cylinder rental. I think that's disgusting. The cylinder is $7.40 a month to rent. The valve on the top which makes the oxygen last longer is $50 odd a month. I think that is very expensive for the mobility*. (Woman in her early eighties with COPD)

Such circumstances limited the individual's capacity for health related decision making and for engaging in other desired pursuits. Some patients prioritised essential treatment options and/or medications based on their understanding of urgency and importance, rather than being guided by health professionals. This meant some prescriptions were not filled or compliance with medication regimes was compromised. For example,

*There are three medications, taken from the pharmacy ... it's not just one kind of tablets but three kinds, and it's very expensive. ... There are times, I have to make a decision whether it's medication, or food, or whatever, pay the bills and that, so and sometimes, we don't have enough money, and then we rely on our children to make up the bills and that, and sometimes we don't fit the medicines, one time we miss it until we get enough money, and then get it the next time*. (Migrant wife carer of a husband with complicated diabetes)

On rare occasions, participants, like the carer above, reluctantly sought financial help from family members, or used birthday/anniversary gift vouchers to ease financial pressures, which was frequently still insufficient to fulfil needs.

Due to financial constraints participants were often unable to follow their management plans or proactively engage in secondary prevention activities (e.g., regular check-ups and seeking timely medical attention).

### Affordability of other things

Affordability also related to participants' ability to pay for additional necessities required for the management of the illness, such as: healthy food, exercise and gym memberships and joining social activities. There was a common view among participants that maintaining a healthy lifestyle is more expensive. For patients with diabetes, preventing (and treating) foot ulcers required additional costs.

*Shoes would be a little more expensive. You have to watch carefully the sorts of shoes that you wear, so you are paying more. I think my last pair of shoes was close to $200. So, footwear can be expensive. You go to a podiatrist, which you probably wouldn't normally do, if you didn't have diabetes*. (Female, in her seventies, with diabetes)

Cost of accessing health services was frequently mentioned by the participants as a source of economic hardship. For example,

*Parking, as you know, in the city is very expensive, $27 or something for three hours or something. So what we do? We rotate the parking there, we go park, and then after an hour or so, then my daughter will go and shift the car round, go and check, and then keep on ... so it's a bit too much for us financially, we are not coping*. (Wife carer of a husband with diabetes)

*For me I can't walk for so long because my legs are sore. And it takes me about 35 to 40 minutes to walk from home to Auburn station. So I can't walk there. And, if I take a taxi it costs me about $8 to $10. So it's too expensive. ... If I have the subsidy, then I can go to Auburn station by taxi, and then I can take a train to the city for medical appointment*. (Migrant female, in her seventies, with diabetes)

Participants reported limiting discretionary spending, cutting back on more expensive, healthier foods and reducing participation in regular exercise programs at a gym. Participants did prioritise expenditure on essential treatment, but a limited financial capacity meant many had to compromise in other areas, such as paying for vacations, home renovations and other activities considered important elements of their quality of life. Additional incidental expenses were amplified by the hardship, resulting in a need to make choices between expenditure for health care and other everyday expenses. This meant that paying even the bare minimum of living costs presented challenges. For example,

*When the house is broken, we like to call somebody to fix, but we can't, because we keep the money for the medicine. So we cannot spend the money to the other thing. Like my car is not very good, broken, but we keep it like this, because we cannot pay everything*. (Migrant wife carer of a husband with diabetes)

Others had to give up leisure activities, hobbies, or cut down associated expenditures, for example,

*I'm a painter, it's my life. But I'm suddenly, the first time in my life, I can't afford...I think twice about buying [painting] materials*. (Man in his late sixties with CHF)

*I don't waste my pension. I don't spend it on things. I don't buy magazines. I don't go to the hair dresser. I don't go out a lot*. (Woman in her seventies with COPD)

Most carers were the spouse of their care recipient (n = 10), and/or living in the same house (n = 13), hence they shared similar concerns to patients regarding their economic hardship. Some carers were concerned about the negative financial impact on the household of taking time off of work to care for a loved one and the burden that transportation costs imposed.

### Factors that influenced economic hardship

Factors that influenced whether participants experienced economic hardship included: eligibility for pensions, other government subsidies or allowances (e.g., pension, carer allowance, health care card or Department of Veterans' Affairs Gold Card, Oxygen subsidy scheme), and/or a concession card (for additional discount rates for electricity, water and other home care services). For example,

*I'm lucky there. The medicines, because I'm on a pension, thankfully, I get them for $4.90. But if I wasn't on the pension, I don't know whether I could afford to pay what some of these medicines cost. I've got a puffer there that's $77, but I get it for $4.90*. (Woman, in her seventies, with COPD)

Most participants who were eligible for pensions or other government subsidy were grateful for use schemes, for example "I'm surviving financially because of the welfare system". However for some, this support was still inadequate to overcome economic hardship and any additional costs not covered by Medicare or other support measures posed an economic burden on their life. For example,

*I walked in there [chemist] last time to get my Webster Packs for a month and I got an unexpected bill for $85 and that was hard. I'm on one or two over the counter medications and they're not funded by Government*. (Man, in his early sixties with CHF and diabetes)

*However desperate my low income situation, I have been warned that the pacemaker will have to be replaced in due course and it would be fool-hardy to attempt stopping my heart drugs in order to redeem my now less-than-basic pension*. (Man in his late sixties with CHF)

For those who were ineligible for pensions or government subsidies, often due to their means tested income exceeding the eligibility criteria, economic hardship was severely felt and was reported to impact significantly on their illness management. For example,

*I mention it to her [doctor], "we can't buy the medicine that you prescribed, because we haven't got money", and she said well "I know, well that's why [your] husband blood sugar level is high, because he's not taking the tablets", because I can't buy them from the pharmacy, so ... and we don't have the card, the concession card that reduces the price of the medication, so what do we do? *(Migrant carer whose husband has diabetes)

*You don't have support anywhere and you are not really highly paid. But you are paid anyway high enough that there is no help ... that was the time that - a few years ago. Now I am actually on a pension and all that. Like, medication side has improved. I had four different medications, so I paid quite a bit around that time and all others and paying rent and all these expenses of the life too. That was when I was working*. (Woman, in her sixties, with diabetes)

In addition, a lack of flexibility in health care services influenced whether participants experienced economic hardship because eligibility criteria were not always inclusive of those most in need. For example, to be eligible for an oxygen subsidy scheme (free, limited, oxygen support for people with COPD) patients would have to be a permanent resident of the ACT. Hardship was exacerbated when patients had 'co-morbidities' or 'multi-morbidities' with the cost of illness management increasing as more illnesses were being managed. The level of health literacy, in terms of patients' and carers' awareness of the system and services, also played an important role in the ability to access subsidies, income support or other available benefits (e.g., free oxygen, community transport or taxi vouchers). Lack of knowledge of self-care added economic hardship, costing both the participants and the health care system. For example, a patient with CHF developed pulmonary oedema and was admitted to hospital due to her lack of understanding of diet. She said,

*Last time when I went into hospital I was drinking four and five cups of Milo a day plus water. I didn't realise Milo was a drink; I thought it was a food*. (Woman, in her eighties, with CHF)

As a consequence of experiencing financial pressures, participants reported the need to be extremely vigilant with expenses and to limit physical and social activities in order to minimise economic hardship and maintain some capacity to act on lifestyle risk factors and balance their life and illness management requirements.

## Discussion

Earlier qualitative studies of chronic illness experiences [27-30] support what has been found in our study regarding the kinds of economic hardship associated with managing chronic illness. These include the individual's compromised ability to afford not only essential treatment and medication but also to maintain a healthy lifestyle and quality of life. Managing serious, long-term conditions requires ongoing financial commitments regardless of the individuals' economic capacity. Despite a health care system that provides universal coverage and a well established and extensive system of social security, individuals with chronic illness still face the long term prospect of economic hardship and severe quality of life impairment. There is evidence in this study that individuals in these circumstances lack adequate resources and support to negotiate and overcome these challenges.

The findings confirm that economic hardship seriously compromises people's healthy lifestyle choices, for instance, due to participants' inability to afford fresh fruit and vegetables that are often more expensive than processed food with higher fat, sugar and sodium content. There appears to be a potential risk that people with chronic conditions, in particular those already exposed to long-term economic hardship, will be more seriously impacted by the global economic downturn. Further research in this area is necessary to investigate any causal relationship with this phenomenon.

This study adds to previous research by identifying a potential risk group for whom the impact of economic hardship on their management of chronic illness was reported as greater relative to others in this study, and which may be jeopardised further by the economic downturn. The groups most at risk include those who are: not in paid employment; on multiple medications; experiencing co-morbidity; from culturally and linguistically diverse (CALD) or Indigenous backgrounds; and/or not eligible for government subsidies and financial support (e.g., low income employees or an income bracket neither sufficiently low for government subsidy eligibility nor high enough to afford necessary expenses; or self-funded retirees without good cash reserves). Interestingly, our study suggests that the problems associated with economic hardship are not geographically specific and are not restricted to locations known to have higher concentrations of residents with lower socioeconomic status (SES). Whilst the ACT population is relatively evenly distributed across SES groupings, with few lower SES pockets [31], a considerably higher number of the ACT participants experienced economic hardship due to costs involved in medical treatment and care. This indicates to policymakers that they cannot assume compensating measures for economic hardship can continue to be based on traditional geographic assessments of SES.

The study highlights the diversity of factors influencing economic outcomes when faced with chronic illness and adds value to the existing literature in the area by offering a qualitative perspective to understanding this issue. Whilst in principle economic hardship can derive generally from either loss of income or costs of care to patients and their carers, such impacts are potentially mediated by multiple individual, community and social levels variables. Indeed, Saunders [15] argues that there are limitations of relying on simply income based measures of economic hardship.

Although not explored in detail by this study, participants discussed some coping strategies to manage their ongoing economic hardship. One strategy discussed was prioritising essential treatments or living expenses, including those related to their care and management. However, participants still had to make a choice between purchasing essential treatments and medications and paying for basic living expenses as most could not afford both. Recent Australian studies provide similar results. Hynd et al. investigated the impact of a co-payment increase for dispensing Australian Government-subsidised medicines and found a significant reduction in the patient's ability to afford essential medicines following the initiative [32]. In the 2008 Commonwealth Fund International Health Policy Survey, 36% of Australian participants (n = 593) reported access problems (accessing physicians, filling prescriptions, or getting recommended test, treatment, or follow-up) because of cost [33]. The Menzies Centre for Health Policy's (MCHP) national survey (n = 1,200) conducted in 2008 confirmed these findings [34]. In addition, the MCHP survey found that high levels of financial stress actually lower individuals' confidence in the system's capacity to provide safe and quality care [34].

Internationally there are an increasing number of studies which report often catastrophic economic consequences associated with chronic illness in low and middle income countries [35-37]. Xu and colleagues in their examination of data from household surveys in 59 countries, argue that middle and low income countries in particular ought to address gaps in their health policy in terms of available health services, health insurance, out-of-pocket payments and financial risk protection to minimise catastrophic health expenditures and potential consequences of impoverishment [37]. The findings in this study indicate the need for further research on the nature of catastrophic health spending among people with chronic, complex conditions and its relationship with health policies in developed countries such as Australia in which such phenomenon is often overlooked.

The main limitations of our study include the lack of generalisability of data obtained from two local areas of Australia, mostly urban; a small sample size and convenient sampling of carer participants. However, the aim of this secondary analysis was to inform the development of policy interventions that are sensitive to the local context and time. Given that participants were mostly older people, some of the economic impacts of chronic illness unique to younger populations--such as a loss of income due to inability to work and its consequences--were not raised as a key issue, hence not fully explored during the interviews. A further study is warranted looking at younger populations. We relied upon the participants' descriptions of their clinical conditions and their management through the interview and the questionnaire. However the participants were recruited through their primary clinicians and we were able to establish the participants had the index condition(s) with moderate to severe symptoms. As a qualitative inquiry, the study focused largely on individual participants' rich descriptions of their experience.

Given the limitations above, caution is necessary in generalising the findings. However, this research fills a gap in the chronic illness literature by providing an understanding of the economic impacts of managing chronic conditions and the hardship that patients and families experience as a result. It provides a platform for further research into strategies to improve the affordability of illness management, particularly self-management activities, given the significant role they play in controlling the progression of illness. In addition, it highlights a need to critically appraise current health, social and welfare policy in order to identify possible options for alleviating hardship. This will require the following:

1. A detailed investigation of the different dimensions of economic hardship experienced by households affected by chronic illness to measure the scope and scale of the hardship, the coping strategies employed to manage and, where possible, to overcome the hardship.

2. An economic evaluation of subsidising secondary prevention measures (e.g., food, exercise and transport) with additional funding for medical costs (e.g., medication, oxygen and health care equipment), in comparison with health care services provision for those who end up in hospital due to failures in existing primary care and social support arrangements.

3. A review of current eligibility criteria for health and social care and other support policies associated with funding subsidies for people with chronic illness, in particular those with multi-morbidity.

It is important to note that the study was undertaken in late 2007 and early 2008, before the major impact in Australia from the Global Financial Crisis (GFC). The economic hardships articulated by interviewees are therefore not simply a result of the deteriorating economic environment; hence suggest a systemic rather than a transient problem in this area. There are mixed theories about the impact of GFC on health in terms of mortality and morbidity rates as well as the health care system's response/reaction (i.e., workforce, health expenditure) [38,39]. The study findings however highlight the negative consequences of economic hardship caused by long-term costs of chronic disease management on the individual's capacity to effectively continue to manage their chronic conditions. In a climate of global economic instability--leading to, for example, increased unemployment, job insecurity, and loss of income--policy makers, health service providers and practitioners need to be more vigilant on issues of care management affordability.

## Conclusion

The study is the first Australian qualitative inquiry that explored the economic impact of common chronic illnesses (COPD, CHF and diabetes) based on the perceptions of patients and family carers. This research provides both insights on the economic stressors associated with managing chronic illness and evidence that such economic hardship requires households to make difficult decisions between care and basic living expenses, which at times compromises necessary treatments and health care. Acknowledging the limitations of the study, the findings underscore the need to consider the cost of lifestyle changes required as part of chronic disease management in developing future policy to address economic hardship among people affected by chronic illness. Further research is necessary to explore the ways in which people cope with economic hardship and prioritise conflicting demands to balance between managing chronic illness and living a normal life. Future research should focus on developing an understanding of health care decisions that are likely to cause less than optimal health outcomes and result in increased costs to the health system.

For health care practitioners these insights are fundamental to providing appropriate and flexible care for chronically ill patients and support for their families. The findings highlight the need to examine in-depth, and even to challenge, common perceptions of the economic impact of chronic illness with respect to geographic or government jurisdictional boundaries. Future policy needs to be multi-sectoral, focusing not only on clinical issues but also on the individual, their household and economic capacity to manage chronic illness.

## Competing interests

The funding organisation (NHMRC) had no role in the study design, data collection, analysis and interpretation, or the writing and publication of this article. The authors declare that they have no competing interests.

## Authors' contributions

Y-HJ conceived and designed the study, participated in all stages of data collection and analysis. Y-HJ drafted and revised the paper as a whole and contributed to revisions and the final version of the manuscript. BE participated in the collection and interpretation of the data. BE, SJ, RW and JW contributed to the early design concepts for the study and actively contributed to the writing and production of the final manuscript. All authors read and approved the final manuscript.

## Pre-publication history

The pre-publication history for this paper can be accessed here:


